# Examining the Causal Structures of Deep Neural Networks Using Information Theory

**DOI:** 10.3390/e22121429

**Published:** 2020-12-18

**Authors:** Scythia Marrow, Eric J. Michaud, Erik Hoel

**Affiliations:** 1 Allen Discovery Center, Tufts University, Medford, MA 02155, USA; scythia@marrow.science; 2 Department of Mathematics, University of California Berkeley, Berkeley, CA 94720, USA; ericjmichaud@berkeley.edu

**Keywords:** artificial neural networks, causation, information theory

## Abstract

Deep Neural Networks (DNNs) are often examined at the level of their response to input, such as analyzing the mutual information between nodes and data sets. Yet DNNs can also be examined at the level of causation, exploring “what does what” within the layers of the network itself. Historically, analyzing the causal structure of DNNs has received less attention than understanding their responses to input. Yet definitionally, generalizability must be a function of a DNN’s causal structure as it reflects how the DNN responds to unseen or even not-yet-defined future inputs. Here, we introduce a suite of metrics based on information theory to quantify and track changes in the causal structure of DNNs during training. Specifically, we introduce the *effective information* (*EI*) of a feedforward DNN, which is the mutual information between layer input and output following a maximum-entropy perturbation. The *EI* can be used to assess the degree of causal influence nodes and edges have over their downstream targets in each layer. We show that the *EI* can be further decomposed in order to examine the sensitivity of a layer (measured by how well edges transmit perturbations) and the degeneracy of a layer (measured by how edge overlap interferes with transmission), along with estimates of the amount of integrated information of a layer. Together, these properties define where each layer lies in the “causal plane”, which can be used to visualize how layer connectivity becomes more sensitive or degenerate over time, and how integration changes during training, revealing how the layer-by-layer causal structure differentiates. These results may help in understanding the generalization capabilities of DNNs and provide foundational tools for making DNNs both more generalizable and more explainable.

## 1. Introduction

Deep neural networks (DNNs) have shown state-of-the-art performance in varied domains such as speech synthesis [1], image recognition [2,3], and translation [4]. These immense advances have been due to the introduction of deep learning techniques [5] to artificial neural networks and the use of GPUs for high-speed computation [6]. Yet the performance of DNNs remains mysterious in multiple ways. For instance, fundamental machine learning theory suggests that models with enough parameters to completely memorize large data sets of images should vastly overfit the training data and lead to poor generalization, especially in models that are not regularized [7]. However, in practice, deep neural networks have good generalization performance, even when not explicitly regularized [8]. While it is well known that artificial neural networks can approximate any given function [9], how the functions they arrive at generalize beyond their training data is less well understood.

One promising approach to explaining the generalization capability of DNNs is the information bottleneck approach [10,11]. The information bottleneck approach conceives of DNNs as optimizing the trade-off between compression of input data into an internal representation and prediction of an output using this representation. Proponents of this approach analyze DNNs by their behavior in the “information plane”, composed of layer-to-input mutual information scores given a data set as input [12]. While looking for information bottlenecks has been a rich research program, larger networks are still plagued by information estimation issues [13], and there have been errors in predictions or deviations for certain network topologies and activation functions [14]. More fundamentally, the information bottleneck approach is in its mathematical formulation data-dependent, that is, its mutual information scores vary with changes to input distributions. Yet generalizability exists for both within-distribution and out-of-distribution data and is definitionally a function of performance across different data sets with different frequencies of inputs, or even unknown and not-yet-defined future data sets. Therefore, to understand generalizability fully it is necessary to focus on what is invariant in DNNs across different data sets with different properties [7].

Examining what is independent across differing data sets means investigating the causal structure of DNNs themselves. That is, uncovering the set of causal relationships (dependencies) between the nodes in the network using techniques from the field of causal analysis. As the causal relationships between nodes are a function of nodes’ mechanisms and connectivity and the dependencies those entail, these relationships are independent of any given data distribution. Information-theoretic techniques can then capture the information contained just in the causal relationships between the nodes (the full set of a layer’s relationships is what we refer to as the causal structure). Here, we introduce a perturbational approach that uses information theory to track the causal influences within a DNN in a layer-by-layer manner. Specifically, we introduce the *effective information* (EI), which captures the informativeness and therefore strength of a causal relationship. The EI was originally introduced as a information-theoretic measure of the causal relationships between two subsets of a complex system [15]. EI has already been shown to quantify the causal structure of Boolean networks [16], and also graphs, by measuring the amount of information contained in the dynamics of random walkers [17]. Notably, EI has mathematical similarities to the information bottleneck approach, although it is focused on causation and therefore differs in key ways.

To measure the EI between feedforward layers of a DNN, we evenly bin the activation range of nodes, inject independent and simultaneous white noise (maximum entropy) into a layer, then calculate the transmitted mutual information to the downstream targets. This captures the total amount of information in the causal structure of that layer-to-layer connectivity. Looking across network architectures, tasks, and activation functions, we observe that steep changes in the loss curve are reflected by steep changes in the EI.

Additionally, EI can be used to track how the causal structures of layers in DNNs change in characteristic ways during training. Specifically, we show how to track DNNs during training in the space of possible causal structures (the “causal plane”), such as whether the connectivity becomes more informationally degenerate or more sensitive. This allows us to show how DNNs develop specific layer-by-layer causal structures as they are trained. We hypothesize that the differentiation of layer-by-layer causal structure may assist generalizability, as networks trained on simpler tasks show less differentiation than those trained on complex tasks, differentiation ceases or slows after the network is fitted to its task, and redundant layers generally fail to differentiate in the causal plane. Additionally, we show how the EI can be used to calculate the difference between the total joint effects and the total individual effects of nodes in a layer, allowing for the measuring of feedforward integrated information in a deep neural network [18].

The tools put forward here to assist in analyzing the causal structures of DNNs using information theory should assist with another central problem of the field, which is that large parameterizations often make DNNs into “black boxes” with millions of fine-tuned weights that allow for successful performance but that are impenetrable in their operations and functions [19]. A lack of explainability can mask other problems, such as biases in either datasets [20] or model choice [21], and is a serious problem for those who want to use DNNs to make life and death decisions, such as in the case of self-driving cars [22], autonomous drones [23], or medical diagnoses [24]. Using this suit of techniques, researchers will be able to directly observe the process during training wherein the overall causal structure of a DNN changes, a key step to opening up the “black box” and understanding what does what in DNNs.

## 2. Quantifying the Causal Structure of DNNs

Interventions (also called “perturbations”) reveal causal relationships. The set of causal relationships (also called the“causal structure”) of a feedforward DNN is composed of layers, their respective connections, and the activation functions of the nodes. We introduce tools to explore the hypothesis that the generalizability of DNNs is a matter of how their causal structures differentiate to fit the tasks they are trained on (all code is publicly available, see https://github.com/ei-research-group/deep-ei).

To investigate this issue, we make use of a formal approach widely used to study causation where interventions are represented as the application of a do(x) operator [25]. The do(x) is normally used to set an individual variable in a given system, such as a directed acyclic graph, to a particular value (for instance, it has been used previously to apply individual interventions in DNNs [26,27]). Rather than tracking individual interventions, in order to generate an analytic understanding of the full causal structure of a DNN layer, we introduce here the use of an intervention distribution, $I_{D}$, which is a probability distribution over the do(x) operator. The $I_{D}$ is simply a mathematical description of a set of interventions. The application of an $I_{D}$ over the inputs of a layer leads to some distribution of effects at the downstream outputs (the $E_{D}$) [28].

The informativeness of a causal relationship can be measured via information theory using an $I_{D}$. More informative causal relationships are stronger. Here, we make use of *effective information* (EI), a measure of the informativeness of a causal relationship, to quantify and examine the causal structure of a layer. Specifically, the EI is the mutual information between interventions and effects, $I(I_{D},E_{D})$, when $I_{D}=H^{\max}$, the maximum-entropy distribution. Put more simply, the EI is the mutual information (MI) following a noise injection in the form of randomization. Note that this randomization serves multiple purposes. First, unlike the standard MI, which is explicitly a measure of correlation [29], all mutual bits with a noise injection will necessarily be caused by that noise. Additionally, as the maximally-informative intervention (in terms of its entropy), EI represents the information resulting from the randomization of a variable, which is the gold standard for causation in the sciences [30], with the number of bits revealing the strength of the causal relationship. Additionally, it can also be thought of as an unbiased sampling of the state-space of an input, meaning that it reflects how the network transmits out-of-distribution data. Finally, the EI can be thought of as measuring how well the image of the function can be used to recover the pre-image, and has important relationships to Kolmogorov Complexity and VC-entropy [31]. Most notably, previous research has shown that EI reflects important properties for causal relationships, capturing how informative a causal relationship is, such as their determinism (lack of noise) or degeneracy (lack of uniqueness) [16], properties which the standard MI does not measure.

First, we introduce a way to measure the EI of layer-to-layer connectivity in a DNN, capturing the total joint effects of one layer on another. Therefore, we start with $L_{1}$, which is a set of nodes that have some weighted feedforward connection to $L_{2}$, and we assume that all nodes have some activation function such as a sigmoid function. In order to measure EI, $L_{1}$ is perturbed at maximum entropy, $do(L_{1}=H^{\max})$, meaning that all the activations of the nodes are forced into randomly chosen states. $L_{1}=H^{\max}$ implies simultaneous and independent maximum-entropy perturbations for all nodes *i* in $L_{1}$:

(1) $$EI=I\left(L_{1},L_{2}\right)\;|\;do\left(L_{1}=H^{\max}\right)$$

That is, the calculation is made by measuring the mutual information between the joint states of $L_{1}$ and $L_{2}$ under conditions of $L_{1}=H^{\max}$.

**Figure 1:**
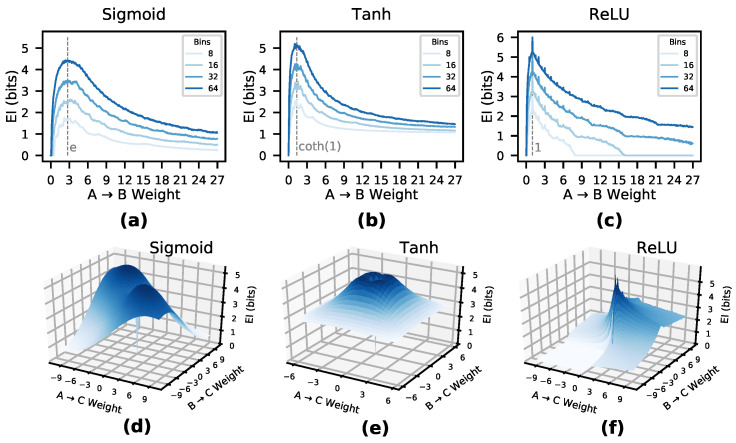
EI is a function of weights and connectivity. Plots (**a**–**c**) show EI vs. weight for a single input and output neuron, using sigmoid, tanh, and ReLU activation functions, and computed using 8, 16, 32, and 64 bins. Marked is the most informative weights (in isolation) for transmitting a set of perturbations for each activation function. Plots (**d**–**f**) show EI for a layer with two input nodes *A*, *B* and a single output nodes *C*. Different activation functions have different characteristic EI manifolds.

EI scales across different commonly-used activation functions. Figure 1a–c shows the EI of a single edge between two nodes, *A* and *B*, wherein A→B with increasing weight, with each panel showing a different activation function (sigmoid, tanh, ReLU). We can see that for each isolated edge with a given activation function there exists a characteristic EI curve dependent on the weight of the connection from *A* to *B*, and that the shape of this curve is independent of the number of bins chosen (8, 16, 32, and 64). At low weights, the EI shows that *B* is not sensitive to perturbations in *A*, although this sensitivity rises to a peak in all three activation functions. The curve then decays as the weight saturates the activation function, making *B* insensitive to perturbations of *A*.

Note that the characteristic peaks reveal which weights represent strong causal relationships (of a connection considered in isolation). For instance, a sigmoid activation function has the most informative causal relationship at a weight equal to Euler’s number *e*, a tanh activation function at weight coth(1), and a ReLU activation function at weight 1. This indicates that the most important weights in a DNN may be the most causally efficacious, not the highest in absolute value. For example, with sigmoid activation functions and an extremely high weight connecting A→B, *A*’s activation is not very informative to perturb, as most perturbations will lead to a saturation of *B*’s output at 1.

In the case of multiple connections, the EI curve becomes a higher-dimensional EI manifold. Figure 1d–f shows the EI(A,B→C) of a layer comprised of two nodes (*A*, *B*) each with a single connection to *C*. As perturbations can interfere with one another, the EI depends not only on the sensitivity of the relationships between nodes, but also the overlap, or degeneracy, of the network connectivity, thus creating a manifold. For instance, in sigmoid activation functions, the EI manifold is roughly 2-fold symmetric, which is due to the symmetric nature of the sigmoid around positive and negative weights, combined with the symmetric nature of the network itself, as both neuron *A* and *B* only connect to *C*.

Note that while the number of bins determines the amplitude of the curve, the rise/decay behavior is consistent across them, indicating that as long as bin size is fixed at some chosen value, ratios and behavior will be preserved (Figure 1 uses 30,000 timesteps for the noise injection for panels (a–c) and 100,000 samples for panels (d–f)). That is, EI values for a DNN layer converge to a particular value if the noise injection is long enough and the bin size is high enough, which contradicts the idea that mutual information in a deterministic system is always infinite ([32]), as this infinity is based on the assumption of an infinite number of bins: given a finite number of bins the EI appears to converge. Evidence for this and more information on EI calculation can be found in the Appendix A.1.

First, however, we assess how changes to EI occur during training networks on common machine learning tasks.

## 3. Information in the Causal Structure Changes During Training

To understand how the causal structures of DNNs change during learning, we tracked the EI in networks trained on two benchmark classification tasks: Iris [30] and MNIST [33]. For Iris, we trained networks with three densely connected layers 4→5→5→3, and for MNIST we used networks with four densely connected layers 25→6→6→5, using sigmoid activation functions and no biases for both tasks. For MNIST, we reshaped the inputs from 28 × 28 down to 5 × 5 and removed examples of digits 5–9 from the dataset so that the final layer has only 5 nodes—this was necessary in order to reduce the computational cost of accurately computing EI. Networks for both tasks were trained with MSE loss and vanilla gradient descent with a learning rate of 0.01. We trained the Iris networks with a batch-size of 10 for 4000 epochs and the MNIST networks with a batch-size of 50 for 500 epochs. We initialized the weights by sampling from the uniform distribution $W_{ij}=U\left(\left[-\frac{1}{\sqrt{{\mathrm{fan}}_{\mathrm{in}}}},\frac{1}{\sqrt{{\mathrm{fan}}_{\mathrm{in}}}}\right]\right)$. For each task and architecture, we perform three runs with distinct initializations. Using the same respective network architectures, we also trained networks with tanh and ReLU activation functions—results can be found in Appendix A.2. To compute EI, we use a fixed noise injection length of $10^{7}$ samples. We found that in our networks, an injection of this length was enough to ensure convergence (see Appendix A.1). Note, however, that wider network layers may require many more samples.

**Figure 2:**
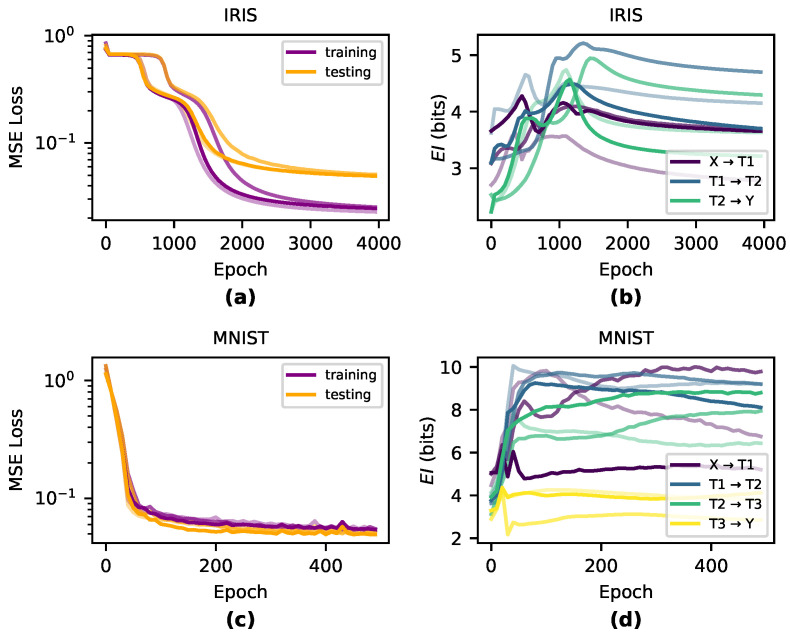
How EI evolves during training across three different runs per condition. Notably, the largest changes in EI occur during the steepest reductions in the loss function for both Iris-trained networks (**a**,**b**) and MNIST-trained networks (**c**,**d**).

Qualitatively, we observe that the greatest changes in EI significantly match the steepest parts of the loss curve during training and EI is generally dynamic during periods of greatest learning (shown in Figure 2). During the overfitting period when training performance dissociated from testing performance, EI was generally flat across all layers, indicating that the information in the causal structure was unchanged during this period after the network had appropriately fitted.

## 4. Deep Neural Networks in the Causal Plane

As discussed in Section 2, EI depends both on the weight of connections as well as their degree of overlap, which together create the EI manifold. This indicates that EI can be decomposed into two properties: the sensitivity of the causal relationships represented by individual weights and the degeneracy of those relationships due to overlap in input weights. This mirrors previous decompositions of the EI in Boolean networks or Markov chains into the determinism (here replaced with sensitivity, since neural networks are traditionally deterministic) and degeneracy [16,17]. This breakdown of EI gives us more information than just whether EI increases or decreases, but shows how the changes to its components lead to changes in the EI, and how EI reveals key properties of a DNN’s causal structure.

In DNNs, the sensitivity of a layer measures how well the input transmits perturbations to the output nodes, while the degeneracy of a layer measures how well the source of input perturbations can be reconstructed by examining the layer output. If the source of a perturbation cannot be reconstructed well the network is said to be *degenerate*. Together, these two dimensions of causal relationships form a “causal plane” which all DNN layers occupy. As layers differentiate via learning, their causal structures should occupy unique positions in the causal plane reflecting their contribution to the function of the DNN by becoming more sensitive or more degenerate.

To identify the position or trajectory of a DNN layer in the causal plane, both sensitivity and degeneracy are explicitly calculated based on the components of EI. The sensitivity is calculated by summing the total contribution of each edge individually, in the absence of interaction effects between parameters. Therefore, the total sensitivity from layer $L_{1}$ to the next layer $L_{2}$ is

(2) $$Sensitivity=\sum_{\left(i\in L_{1},j\in L_{2}\right)}I\left(t_{i},t_{j}\right)\;|\;do\left(i=H^{\max}\right)$$

This is the same as calculating the EI of each (*i*,*j*) pair, but done independently from the rest of the network. Note that in a layer wherein each node receives only one unique input (i.e., no overlap) the sensitivity is equal to the EI.

**Figure 3:**
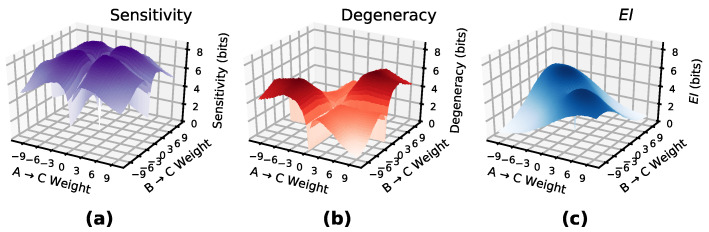
EI is composed of sensitivity and degeneracy. The above surfaces are the sensitivity and degeneracy of a layer with two input nodes and a single output nodes, with a sigmoid activation function. Subtracting the surface in panel (**b**) from the surface in panel (**a**) gives the EI manifold as in panel (**c**).

The degeneracy of a layer measures how much information in the causal relationships is lost from overlapping connections, and is calculated algebraically as sensitivity−EI, as sensitivity measures the information contribution from non-overlapping connections in the network. Figure 3 shows sensitivity and degeneracy manifolds for a layer of two input nodes and one output node (with sigmoid activations) with varying connection weights. The difference between them creates the EI manifold.

Previous research investigating the EI of graphs (based on random walk dynamics) has led to a way to classify different canonical networks, such as Erdős-Rényi random graphs, scale-free networks, and hub-and-spoke models, based on where they fall in terms of the determinism and degeneracy of random walkers [17]. For EI in DNNs a sensitivity term takes the place of determinism.

In order to visualize layer shifts between sensitivity and degeneracy we introduce the “causal plane” of a DNN, wherein the two dimensions of the plane represent the two respective values. The causal plane makes use of the fact that, as EI=sensitivity−degeneracy, if both increase equally, the EI itself is unchanged. When degeneracy vs. sensitivity is plotted, points on the line y=x represent zero EI, and we refer to this $45^{\circ }$ line as the “nullcline” of the EI. Paths that move more towards sensitivity will increase EI, and paths that move more towards degeneracy will decrease EI, while paths along the EI nullcline will not change EI.

**Figure 4:**
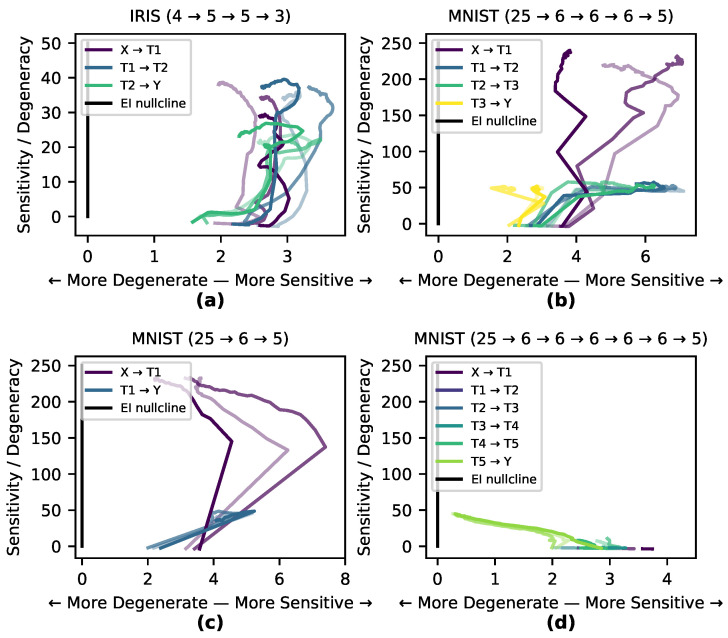
Behavior on the causal plane during training. Paths traced on the causal plane in different layers. All paths get less smooth over time during the period of overfitting and move about less in the causal plane. Networks trained on the simpler Iris task (**a**) show less differentiation between layers than those trained on the MNIST task (**b**). The causal plane shows which layers are redundant, as an MNIST-trained network with a single hidden layer shows significant movement (**c**) whereas for an MNIST-trained network with five hidden layers, all five layers show minimal movement in the plane (**d**).

Here, we explore the hypothesis that the internal causal structure of a DNN shifts to match the task it is trained on, and that this happens in specific stages throughout the training process. To investigate this, we measured the paths of three runs on the Iris and MNIST data sets through the causal plane during training (shown in Figure 4a–b). Of the two tasks, classifying MNIST digits is more degenerate and complex, as the network must transform a manifold in a high-dimensional space into only 10 distinct output classes (or rather 5 for our reduced version of MNIST here). The task of classifying Iris flowers is not as degenerate nor complex, as the network must transform a 4-dimensional space into three (mostly) linearly separable classes. If a network learns by matching its internal causal structure to the data set a network trained on MNIST would shape itself to a greater degree than one trained on Iris. This is precisely what we observe in Figure 4 wherein the MNIST-trained network shows much greater differentiation and movement within the causal plane, while there is less differentiation in the causal structure of the Iris-trained network as it follows the EI nullcline. In many cases, particularly for hidden and output layers, the runs first demonstrate an increase in sensitivity (increasing the EI), and then later an increase in degeneracy (decreasing the EI).

In order to examine the hypothesis that the causal structure of layers necessarily differentiate in response to training, the MNIST-trained network with sigmoid activation functions was modified in two ways: in one case a hidden layer was removed, and in the other case a number of redundant hidden layers were added (Figure 4c–d). Both modifications of the network trained as accurately as the previous network. In the causal plane the added redundant layers moved very little, indicating a net-zero contribution to the EI during training (for movie see https://github.com/ei-research-group/deep-ei). This shows how redundant layers that don’t contribute to the network’s causal structure cluster along the EI nullcline and move little, compared to more dynamic layers.

## 5. Measuring Joint Effects of Layer-to-Layer Connectivity

Integrated Information Theory (IIT) has been used to assess the total information contained in joint effects versus their independent effects in systems [34]. It is a useful tool for causal analysis, analyzing the amount of information being integrated in a network’s causal structure [35,36]. Previously, the integrated information has been measured as the loss in EI given a partition [37], making EI the upper bound for integrated information. However, there is no one accepted and universal measure of integrated information [18,38]. Instead, various measures for integrated information have been put forward in different systems [39,40]. Traditionally, the amount of integrated information in a feedfoward network is zero as there is no reentrant connectivity, as it is based on finding the minimum information partition across all possible subsets of a system. However, even in a feedforward network a layer’s nodes can still contain irreducible joint effects on another layer, and therefore we introduce a measure, feedforward integrated information, to apply in DNNs.

Normally calculating the integrated information requires examining the set of all possible partitions, which prohibits this method for systems above a small number of dimensions. Alternatively, in order to assess the synergistic contribution to EI of individual edges, one would likely need to use multivariate information theory, such as the partial information decomposition, which grows at the sequence of Dedekind numbers as sources are included [41].

In order to avoid these issues we introduce a measure, $EI_{parts}$, which is calculated based on contributions of each edge. That is, for each node $i\in L_{1}$ a sample $t_{i}$ of its activation function under a maximum-entropy perturbation is recorded, along with that of each node $j\in L_{2}$. To calculate $EI_{parts}$, each sample of each node is discretized into some shared chosen bin size, and the MI of each (*i*,*j*) pair is calculated and summed:

(3) $$EI_{parts}\left(L_{1}\to L_{2}\right)=\sum_{\left(i\in L_{1},j\in L_{2}\right)}I\left(t_{i},t_{j}\right)\;|\;do\left(L_{1}=H^{\max}\right).$$

Note that for a layer with a single node, EI and $EI_{parts}$ are identical. The same is true when each node of the network only receives a single edge. However, $EI_{parts}$ measure will necessarily miss certain positive joint effects. Importantly, the difference between EI and $EI_{parts}$ measures can capture the amount of joint effects, and therefore the amount of information the layer-to-layer is integrating in a feedforward manner. Specifically, we compare EI, the upper bound for integrated information, to $EI_{parts}$ as defined in Section 3, that is $\phi_{feedforward}=EI-EI_{parts}$. It should be noted that $\phi_{feedforward}$, while designed to capture total joint effects of one layer to another, is not bounded by zero and can be negative. The sign of $\phi_{feedforward}$ determines if a network’s higher-order joint effects are informative or noisy. A network with a positive value of $\phi_{feedforward}$ will contain mostly informative joint effects, while a network with a negative value of $\phi_{feedforward}$ will contain mostly noisy joint effects. Note that its possible negative value makes it conceptually similar to the Interaction Information based on information decomposition [42].

**Figure 5:**
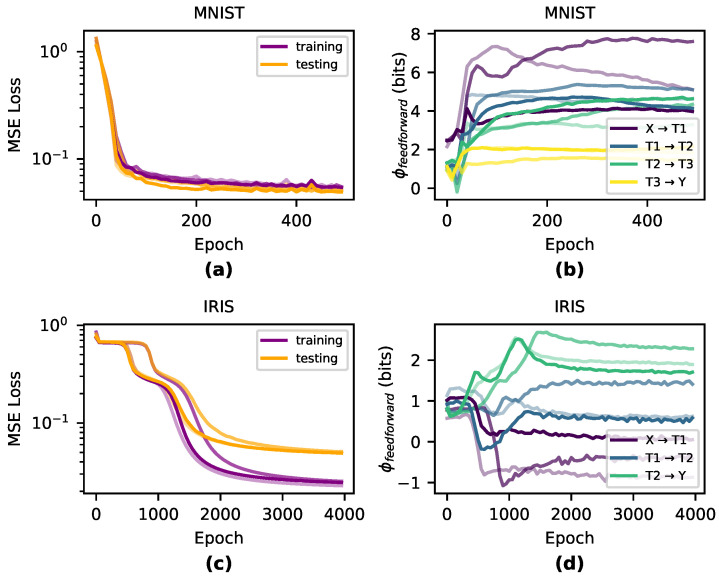
Integrated Information over training. MNIST-trained networks (**a**,**b**) develop more $\phi_{feedforward}$ during training than IRIS-trained networks (**c**,**d**).

To understand how layer-to-layer joint effects change during training of a DNN, we analyzed how $\phi_{feedforward}$ changes during training across both Iris and MNIST data sets (see Appendix A.1 for details on our methodology for measuring $EI_{parts}$). We observe that MNIST-trained networks have higher $\phi_{feedforward}$ than Iris-trained networks, indicating that the causal structure has indeed differentiated in accordance with the complexity of the task and requires more joint effects to learn (Figure 5). This is likely because MNIST requires a more complex network than Iris and requires learning joint effects instead of the more linear learning for Iris.

## 6. Discussion

Here, we have introduced information-theoretic techniques to categorize and quantify the causal structures of DNNs based on information flows following perturbations. These techniques are built around the *effective information* (EI), which we adapted to apply to DNNs. It is defined as the mutual information following a set of perturbations of maximum entropy, and it reveals the information contained in the causal structure of a layer. For networks trained on both Iris and MNIST tasks, EI changed during the training period, particularly when learning actually occurred (as reflected by step changes in the loss function).

EI depends on both the sensitivity and degeneracy of a network. The sensitivity between two nodes reflects the strength of causal relationships in isolation and peaks at particular characteristic weights for different activation functions (e.g., in sigmoid activation functions it peaks at *e*). The degeneracy of a layer reflects the difficulty of downstream reconstruction of an upstream perturbation due to overlap of edge weights. Analyzing the EI reveals where networks lie on sensitivity/degeneracy space, which we call the “causal plane.” The ability to place network architectures in this plane means we can track how any given DNN’s causal structure evolves during its training as it moves through the space. Our results indicate that the causal structure of an DNN reflects the task it is trained on. For instance, in the MNIST task, different layers have a clear task in the causal structure of the DNN, reflected by each layer’s different trajectory in the causal plane, and adding new redundant layers added no new information to the causal structure by not contributing to the EI.

These techniques offer a different approach than work on information bottlenecks [43], which is focused on using the mutual information to measure correlations between inputs and node activity. Both approaches have a similar goal to explain DNN generalizability and both share formal similarities, although here the focus is on the layer-by-layer causal structure itself rather than the input of DNNs. In the future, this work can be extended to different activation functions beyond the three considered here [44,45], unsupervised tasks [46], recurrent neural networks such as LSTMs [47], and convolutional neural networks [2].

These techniques open up the possibility of assessing decompositions and expansions of the EI, such as the integrated information of DNNs (as integrated information can be calculated using the minimum of EI between subsets of a network [15]), and integrated information is also decomposable into properties similar to sensitivity and degeneracy [48]. Here, a measure of integrated information, $\phi_{feedforward}$, is outlined that measures the irreducible joint effects in feedforward layer connectivity.

All of these may help understand why certain network architectures generalize and why some do not. In the future, these techniques also open the possibility for direct measurement of individual instances of causation in DNNs [36].

## Figures and Tables

**Figure A1:**
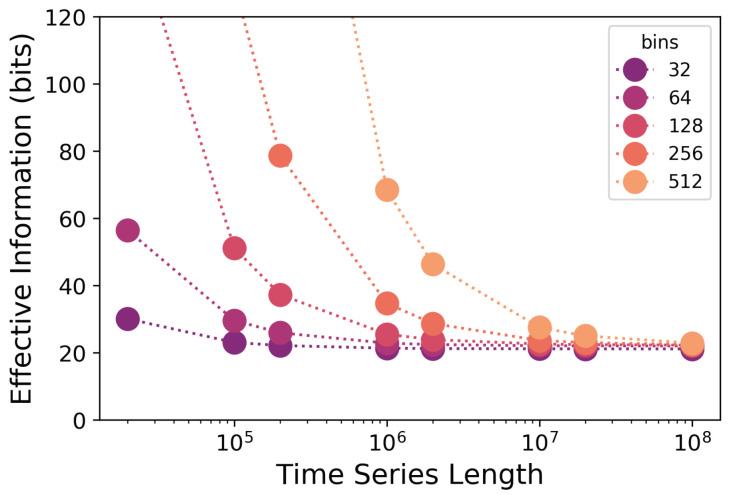
Convergence of $EI_{parts}$ measures to theoretical values. The $EI_{parts}$ a 30→30 layer injected with a sample of noise up to $10^{8}$ time-steps and analyzed with different numbers of bins.

**Figure A2:**
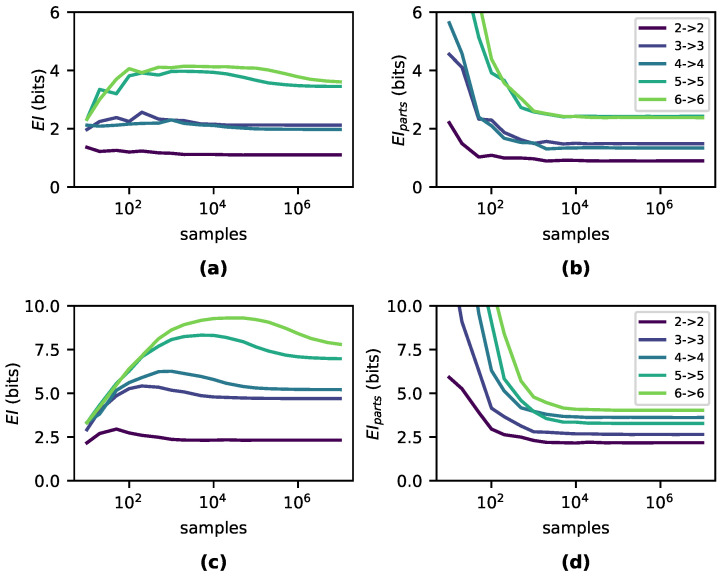
Convergence of EI and $EI_{parts}$. If evaluated on enough noise samples, EI and $EI_{parts}$ converge. In panels (**a**,**b**), we show how EI and $EI_{parts}$, respectively, converge for dense layers of varying width, initialized with the distribution $U(\left[-\frac{1}{\sqrt{{\mathrm{fan}}_{\mathrm{in}}}},\frac{1}{\sqrt{{\mathrm{fan}}_{\mathrm{in}}}}\right])$. In panels (**c**,**d**), we show the same, but with weights sampled from $U(\left[-\frac{5}{\sqrt{{\mathrm{fan}}_{\mathrm{in}}}},\frac{5}{\sqrt{{\mathrm{fan}}_{\mathrm{in}}}}\right])$.

**Figure A3:**
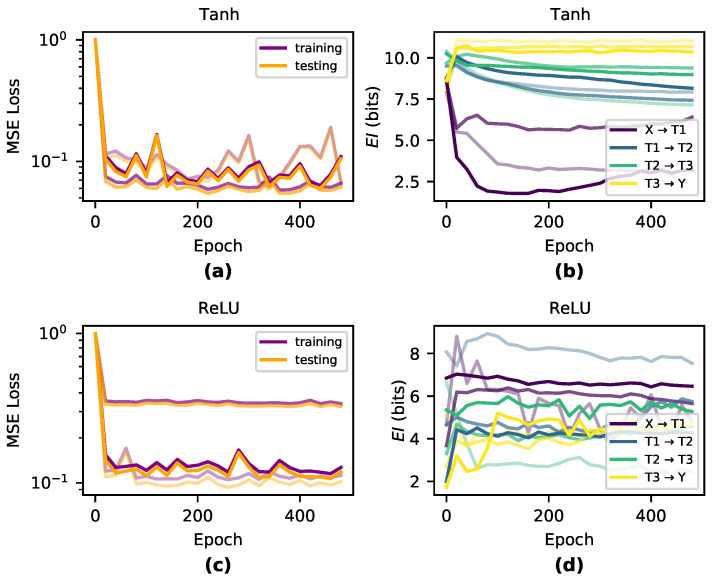
Changes in EI during training across activation functions. Tanh (**a**,**b**) and ReLU (**c**,**d**) versions of a network trained on the reduced-MNIST task, three runs each, showing the different layers.

## Appendix A

### Appendix A.1. Effective Information Converges Across Measurement Schemes and Can Be Found via Extrapolation

Consider a neural network layer consisting of *n* input neurons with feedforward connections to *m* downstream output neurons ($R^{n}\to R^{m}$). EI is defined as the mutual information between layer input and output following a maximum-entropy perturbation. We estimate EI first by uniformly subdividing the range of the chosen activation function (e.g., (0,1) for sigmoids) into *B* bins. Then, an input sample is constructed by independently sampling values uniformly from the activation function range (e.g., (0,1) for sigmoids) for each input neuron. This vector of activations $\in R^{n}$ is then input (“injected”) into the layer, and the output vector $\in R^{m}$ of activations is recorded. Using the binning scheme, the input sample and corresponding output are each converted from a continuous value into a discrete symbol (there are $B^{n}$ symbols in the input space and $B^{m}$ symbols in the output space), and added to a 2d histogram of input–output pairs. After a number of samples are injected, discretized, and added to the histogram, the histogram can be normalized, giving a joint distribution over layer inputs and outputs, from which the mutual information is computed.

$EI_{parts}$ is computed similarly, except instead of building up one large histogram with $B^{n}B^{m}$ entries, nm histograms are computed, one for each pair of input and output neurons, each histogram with $B^{2}$ entries. Each histogram represents a joint distribution between the activations of an input neuron and an output neuron. When $EI_{parts}$ is computed, the mutual information of each pair of input and output neurons is computed from their corresponding histogram, and these nm mutual information values are summed. *Sensitivity* is computed similarly to $EI_{parts}$, except that when calculating the joint distribution between input neuron *i* and output neuron *j*, instead of all input neurons taking random values, only neuron *i* outputs random values, with all other input activations set to 0.

Note, however, that these techniques require a choice both of the number of noise samples used and of the number of bins. In Figure A1, we examined how $EI_{parts}$ converges for a 30→30 dense layer with varying number of bins. The layer was initialized with the uniform distribution from earlier (Section 3). As we see in Figure A1, provided enough bins are used, $EI_{parts}$ generally converges to about the same value regardless of the exact number of bins used. However, the number of noise samples which must be injected for the $EI_{parts}$ to converge greatly increases with the number of bins. With 256 bins, convergence of $EI_{parts}$ can sometimes take millions of samples, and one must therefore be careful about specifying a precise number of samples to use when computing $EI_{parts}$.

To accurately compute $EI_{parts}$ without having to specify a fixed number of samples, we used two techniques. When it was computationally tractable (which it was for all the experiments presented here), we successively double the number of samples used in the injection until the expected change (computed with secant lines through the $EI_{parts}$ vs. samples plot) in $EI_{parts}$ of another doubling is less than 5% of the most recently computed value. In some scenarios, this technique, which computes EI directly, requires many millions of samples (or as many as are needed for the $EI_{parts}$ vs. samples line to level off), and therefore is often intractable for large densely-connected layers, or when a large number of bins are used. As a more tractable alternative, for the larger layers (like those in our MNIST-trained networks) we introduced a way to measure $EI_{parts}$ with varying numbers of samples and fit a curve to the $EI_{parts}$ vs samples relationship. Across a range of layer connectivities and sizes, we observe that the $EI_{parts}$ vs. samples curve takes the form

$$EI_{parts}\left(s\right)=\frac{A}{s^{\alpha }}+C$$

To extrapolate $EI_{parts}$, we evaluate $EI_{parts}$ directly on 100K, 200K, …, 2M samples, then fit the above curve, and evaluate it at $10^{15}$. While this method does not compute $EI_{parts}$ directly, we find that in practice it gives accurate values.

Note that these methods apply only to the computation of $EI_{parts}$ which we find to be monotonically decreasing in the number of samples used to compute it. Computing the full EI is in general a much harder problem. Figure A2 shows convergence curves for both EI and $EI_{parts}$ for layers of varying width, computed with 8 bins per node. As the number of samples used increases, EI at first increases before decreasing and leveling off by $10^{7}$ samples in layers of width no greater than 6 neurons.

### Appendix A.2. Effective Information Tracks Changes in Causal Structure Regardless of Activation Function

Causal relationships should depend on activation functions. To test this, we further examined the EI of Iris and MNIST-trained networks, yet with tanh and ReLU activation functions (shown in Figure A3). Despite using different initializations, training order, and activation functions, the changes in EI during training were broadly similar, although each choice of activation function changed precise behavior in EI.
